# Pain and Successful Aging Among Canadian Adults

**DOI:** 10.1093/geroni/igaf122.074

**Published:** 2025-12-31

**Authors:** Merita Limani, Anna Zajacova

**Affiliations:** Western University, London, Ontario, Canada; Western University, London, Ontario, Canada

## Abstract

Chronic pain, highly prevalent among older adults, is associated with worse health. However, little is known about its impact on successful aging (SA). SA is a multidimensional concept encompassing not only physical and psychological health, but also social connectedness and functional independence in later life. This study examines the impact of chronic pain on SA over time and identifies demographic and socioeconomic factors that may buffer or exacerbate their association. Data are from the Canadian Longitudinal Study on Aging (CLSA), a 3-wave panel study including 30,097 adults aged 45 and older. The primary outcome is SA, which we constructed from 6 domains with indicators collected identically at all waves. We estimate mixed-effects models of SA as a function of pain severity (no pain, mild/moderate pain, severe pain). Models control for age, gender, marital status, race/ethnicity, immigrant status, education, and income, and include appropriate interactions to test buffering. Initial results indicate that chronic pain is associated with lower SA scores, with more severe pain linked to lower baseline scores and greater declines over time. Among covariates, income is particularly salient as a moderator of the association. These findings highlight that chronic pain undermines multiple dimensions of wellbeing essential to successful aging, extending beyond physical and mental health. The significant role of income highlights socioeconomic disparities in pain experiences, suggesting that addressing these inequities could enhance successful aging trajectories.

